# 40 Years of Cracking the Orthographic Code: A Special Issue in Honour of Jonathan Grainger’s Career

**DOI:** 10.5334/joc.413

**Published:** 2024-12-13

**Authors:** Joshua Snell, Sebastiaan Mathôt, Mathieu Declerck

**Affiliations:** 1 Vrije Universiteit Amsterdam, The Netherlands; 2 Rijksuniversiteit Groningen, The Netherlands; 3 Vrije Universiteit Brussel, Belgium

**Keywords:** Reading, Bilingualism, Attention, Mathematical modelling, Semantics, Sentence processing

## Abstract

Anyone recounting the history of cognitive psychology will have to make early mention of the study of orthographic processing (starting in 1886 with the seminal work of Cattell, a doctoral student of Wilhelm Wundt); and anyone recounting the study of orthographic processing will have to make mention of Jonathan Grainger. An honorary member and former president of the European Society for Cognitive Psychology, Jonathan has dedicated nearly four decades of research to the mechanisms driving the recognition of letters, words and sentences during reading. In honour of Jonathan’s career—which formally has come to a close in 2023—in this Special Issue several contemporaries and close collaborators highlight important advances that have been made in the past 40 years, and provide flavours of where the field stands today.

To reading researchers it will come as no surprise that this Special Issue is quite multi-faceted: articles in this issue cover the processing of visual features and letters (Perea, Romero-Ortells, Labusch, Fernández-López, & Marcet, 2023), phonology (Holcomb, Akers, Midgley, & Emmorey, 2024; Magnuson, You, & Hannagan, 2024), morphology (Kahraman & Beyersmann, 2024), semantics (Gavard & Ziegler, 2024) and syntax (Gavard & Ziegler, 2024; Seijdel, Stolwijk, Janicas, Snell, & Meeter, 2024; Snell & Melo, 2024); as well as attention (Snell & Melo, 2024), statistical learning (Gavard & Ziegler, 2024) and multi-lingualism (Kahraman & Beyersmann, 2024; Vandendaele, Prutean, & Declerck, 2024). As such, this Special Issue is nearly as multi-dimensional as reading itself. At the same time—and astonishingly so—the range of topics covered by this Special Issue is easily matched by the breadth of Jonathan’s work. Let us substantiate this claim with a brief review of Jonathan’s career below.

Many careers in cognitive science are characterized by specialization in a single subdomain or methodology. This is certainly also true in reading research: indeed, given the wide variety of topics listed above, surely no sane mind would dare to ‘do it all’? Without putting Jonathan’s sanity into question, he is one of the few exceptions to the rule. At the start of his career, in the ‘80s, Jonathan’s main focus was on bilingualism, where he made observations about cross-language interactions in the brain (e.g., Grainger & Beauvillain, 1987) which were later captured in the Bilingual Interactive-Activation Model (Grainger & Dijkstra, 1992; Dijkstra, van Heuven & Grainger, 1998). These important contributions to our understanding of the bilingual brain remain well-cited to this day and continue to form the basis for new models of bilingual language comprehension (e.g., Dijkstra et al., 2019).

Although he never completely turned his back on bilingualism (see, e.g., recent work by Declerck, Grainger and Hartsuiker (2021)), towards the end of the ‘80s Jonathan broadened his focus. At that time the seminal Interactive Activation Model (IAM) of McClelland and Rumelhart (1981) was steadily continuing to engrave its marks upon history; and Jonathan, together with his mentor Juan Seguí, delivered some of its most crucial tests—in particular relating to the contentious issue of lateral inhibition among lexical representations (e.g., Grainger, O’Regan, Jacobs, & Seguí, 1989; Grainger, 1990).

By the turn of the decade the field had embraced the principle that a cognitive process cannot be fully understood without viewing it in relation to other cognitive processes: this is the essence of *interactive activation* in the IAM. The word superiority effect (Cattell, 1886) was cognitive science’s very first example of this, showing that letter recognition is modulated by lexical activation. While the IAM captures word-to-letter feedback, Jonathan also found plenty of applications of the same principle outside the IAM’s scope. For instance, together with his PhD student Ludovic Ferrand, Jonathan revealed interactions between orthographic and phonological processing (e.g., Ferrand & Grainger, 1992). The contribution by Holcomb et al. (2024) to this Special Issue attests to the field’s enduring interest in phonology’s role in reading.

Although the interactive activation principle has survived to this day, in the ‘90s there also amassed a bulk of evidence against the IAM’s rigid letter position coding scheme. Several years before, Forster and Davis (1984) had pioneered the masked priming paradigm, and throughout the ‘90s Jonathan and many of his peers exploited this technique extensively. One important conclusion was that letter position coding is flexible (e.g., ‘rcok’ primes ‘rock’ more than does ‘rsak’), and this need for a more flexible mechanism started the ongoing quest to—in Jonathan’s words—‘crack the orthographic code’ (e.g., Grainger, 2008).

The fruits of this endeavour were largely borne in the next decade. And these fruits came in many different flavours, such as spatial coding (e.g., Davis, 2010), noisy slot-based coding (e.g., Gomez, Ratcliffe & Perea, 2008) and split fovea processing (e.g., Shillcock, Ellison, & Monaghan, 2000). Jonathan’s bet was on *bigrams*: an intermediate level of representation, in between letters and words, that conveys the relative positions of pairs of letters (Grainger & van Heuven, 2003; see also Whitney, 2001). Although the specifics of such intermediate representations are not yet set in stone, there is now a good deal of evidence to suggest that the literate brain represents not only single letters and words, but also letter combinations (see, e.g., Snell, 2024, for a recent review).

Speaking of letter combinations, Jonathan did not only put bigrams on the map: he also contributed a good deal to our understanding of morphological processing. Though having been limited to a few (albeit well-cited) papers in the ‘90s, by the turn of the millennium this line of inquiry shifted into the next gear in collaboration with PhD student Hélène Giraudo and peers Ram Frost and Kathleen Rastle (e.g., Frost & Grainger, 2000; Giraudo & Grainger, 2001; Frost, Grainger & Rastle, 2005), and later with Elisabeth Beyersmann (e.g., Grainger & Beyersmann 2017; Grainger, Snell, & Beyersmann, 2021). Fully mapping out the morpheme and its operations remains a challenge (both theoretically and empirically); but great strides are being made to this day, as showcased by the contribution of Kahraman and Beyersmann (2024) to this Special Issue.

Before his well-earned retirement, Jonathan still had a decade to fill with research in yet another subdomain: sentence reading. When one studies the recognition of single isolated words, the distribution and dynamics of visuo-spatial attention may not seem worth considering (and indeed, classic word recognition models do not involve attention); but this changes completely when studying the recognition of entire sentences. There had been many others who studied attention and oculomotor control in sentence reading (see e.g. Rayner, 1998, for a review), but those researchers were not primarily interested in the brain’s interface between letters and words. In retrospect, the field’s pronounced division between those interested in eye movements in text reading and those interested in single word recognition may seem odd and unnatural. But it so happened that many phenomena could be explained within one subdomain without consideration for the other; and thus everybody had been content. This changed when it was decided to expand Jonathan’s Open Bigram model (Grainger & van Heuven, 2003) into the realm of text reading. For reasons not at all pertaining to intergalactic space battles the new model was coined OB1-reader (Snell, van Leipsig, Grainger, & Meeter, 2018), and many exciting debates ensued. For instance, from an ‘Open Bigram perspective’ it was a natural theoretical decision to assume that orthographic information is pooled from multiple words in parallel; but doing so implied a direct contradiction of the well-established model of text reading E-Z reader, which assumes strictly serial word processing (Reichle, Rayner, & Pollatsek, 2003). And if multiple words can be processed in parallel, how does the brain keep track of word order? The solution was a sentence-level representation onto which activated words are mapped, guided by visual cues and contextual constraints. It was also time to invoke the interactive activation principle once again: analogous to how letter activation is modulated by word representations, it was hypothesized that word activation is modulated by syntactic (sentence-level) representations. As such OB1-reader correctly predicted readers’ tendency to fail to notice the error in ‘Do love you me?’ (e.g., Mirault, Snell, & Grainger, 2018) and the existence of sentence superiority effects (i.e., faster recognition of ‘man’ in ‘the man can run’ than in ‘run man the can’; e.g., Snell & Grainger, 2017). Thanks to Jonathan, there is currently much interest for the mechanisms underlying (flexible) word position coding (see, e.g., Hossain & White, 2023; Snell, *in press*). The contribution by Snell and Melo (2024) in this Special Issue provides a flavour of this ongoing debate, and the contribution by Seijdel et al. (2024) showcases current computational modelling endeavours to capture sentence-to-word feedback.

Here we have provided but a coarse chronology of Jonathan’s career. In addition to the themes highlighted above there were various other topics that recurred regularly, including reading development (e.g., Grainger, Bertrand, Lété, Beyersmann, & Ziegler, 2016; Snell, Cauchi, Grainger, & Lété, 2021), dyslexia (e.g. Ziegler, Pech-Georgel, Dufau, & Grainger, 2010) and even reading in non-human primates (e.g., Grainger, Dufau, Montant, Ziegler, & Fagot, 2012). If there is one key defining aspect of Jonathan’s career, then, it is that no stone has been left unturned. But we would be remiss if we did not mention a second key defining aspect of Jonathan’s career: namely, that it was a true joy for any PhD-student, post-doc or peer to be part of the journey. All who have had the pleasure of working with Jonathan, whether briefly or extensively, will agree with us that Jonathan was one of the kindest people on and off the work floor. He always ensured an open and inspiring atmosphere, and, in spite of his own respected status, always dedicated himself to helping others succeed.

For a scientist of Jonathan’s calibre there is probably never a perfect time to call it quits: the more domains you’re invested in, the more likely your retirement is to entail an untimely abdication from new, exciting and unsolved debates. Indeed, as we believe is adequately conveyed by this Special Issue, the study of orthographic processing is now more multi-dimensional than ever before; and much of this is owed to one very multi-dimensional man.
